# Growth on Metallo-Supramolecular Coordination Polyelectrolyte (MEPE) Stimulates Osteogenic Differentiation of Human Osteosarcoma Cells (MG63) and Human Bone Marrow Derived Mesenchymal Stem Cells

**DOI:** 10.3390/polym11071090

**Published:** 2019-06-27

**Authors:** Janina Belka, Joachim Nickel, Dirk G. Kurth

**Affiliations:** 1 Lehrstuhl für Chemische Technologie der Materialsynthese, Universität Würzburg, Röntgenring 11, D-97070 Würzburg, Germany; 2 Lehrstuhl für Tissue Engineering und Regenerative Medizin, Universitätsklinikum Würzburg, Röntgenring 11, D-97070 Würzburg, Germany; 3 Fraunhofer ISC, Translationszentrum für Regenerative Therapien (TLZ-RT), Röntgenring 11, D-97070 Würzburg, Germany

**Keywords:** cell differentiation, metallo-supramolecular polymer, interface, iron metabolism

## Abstract

Background: Culturing of cells is typically performed on standard tissue culture plates generating growth conditions, which in general do not reflect the native three-dimensional cellular environment. Recent investigations provide insights in parameters, which strongly affect the general cellular behavior triggering essential processes such as cell differentiation. The physical properties of the used material, such as stiffness, roughness, or topology, as well as the chemical composition of the cell-surface interface are shown to play a key role in the initiation of particular cellular responses. Methods: We extended our previous research, which identified thin films of metallo-supramolecular coordination polyelectrolytes (MEPEs) as substrate to trigger the differentiation of muscular precursor cells. Results: Here, we show that the same MEPEs similarly stimulate the osteogenic differentiation of pre-osteoblasts. Remarkably, MEPE modified surfaces also trigger the differentiation of primary bone derived mesenchymal stem cells (BMSCs) towards the osteogenic lineage. Conclusion: This result leads to the conclusion that these surfaces individually support the specification of cell differentiation toward lineages that correspond to the natural commitment of the particular cell types. We, therefore, propose that Fe-MEPEs may be used as scaffold for the treatment of defects at least in muscular or bone tissue.

## 1. Introduction

In a previous study, we investigated the influence of Fe-MEPE modified surfaces on the differentiation of pre-myoblastic C2C12 cells in standard cell culture medium [1]. C2C12 cells differentiate towards myotubes after being seeded onto Fe-MEPE modified surfaces without the need to add agents that promote differentiation. In case of C2C12 cells, if grown on standard cell culture plates, myogenic differentiation is typically initiated by serum starvation [2]. To verify the hypothesis that culturing cells on Fe-MEPE modified surfaces might in general stimulate cell differentiation towards pre-determined differentiation lineages, we extended our investigations on differentiation of the human osteosarcoma cell line MG63 and that of primary human bone marrow derived mesenchymal stem cells (BMSCs).

Using standard cell culture plates differentiation of MG63 cells generally requires addition of special agents such as ascorbic acid and dexamethasone [3,4]. Standard protocols for the osteogenic differentiation of primary pluripotent BMSCs also utilize dexamethasone and ascorbic acid, but may also require addition of bone morphogentic protein (BMP)2 and β-glycerophosphate [5]. Dexamethasone primarily promotes cell proliferation whereas ascorbic acid induces expression of alkaline phosphatase (ALP) and osteocalcin [6,7]. Despite of their osseous origin, BMSCs can be differentiated towards different lineages in vitro such as osteoblasts, chondrocytes, astrocytes, neurons, skeletal, and cardiac muscles [8,9,10,11]. Even though these cells are pluripotent, they are committed to differentiate towards bone cells.

As a general sign of differentiation, cellular parameters such as cell growth and mitochondrial activities are assessed since high mitochondrial activities at low cell count indicates cell differentiation. The osteogenic differentiation process is characterized by several steps. At first, the cells adhere and proliferate. Subsequently, the extracellular matrix is formed, which is finally followed by mineralization. Collin et al. considered clustering of cells before matrix formation as a separate step [12].

During the formation of extracellular matrix the osteoblasts produce collagen type I, osteocalcin, osteopontin, and alkaline phosphatase (ALP), which drastically increases its activity at the beginning of bone matrix mineralization [13]. The high activity of ALP leads to an increased release of phosphate, which forms the mineral part of the bone with free calcium ions [14]. At onset of osteogenic differentiation, ALP is up-regulated, whereas osteocalcin is primarily expressed at a later stage upon mineralization. According to Collin et al., ALP is expressed at the beginning and at the end of these differentiation processes [12]. Owen et al. assume that ALP increases linearly during differentiation and drastically increases before mineralization [15]. With the beginning of mineralization, osteocalcin reaches its maximum of expression [16]. According to Bronckers et al. increased osteocalcin expression occurs in osteoblasts and osteocytes [17].

However, it is known that primary cells do not differentiate synchronously in vitro [18]. Thus, in a pool of cells, both ALP and osteocalcin expression may be detected at the same time. Additionally, cell differentiation is dependent on the site of cell collection, the number of residing cells, the gender of the donor, and on cell extraction and purification methods [19]. However, a detailed understanding of the orchestrated interaction and regulation of operating signaling pathways and regulating factors involved in these differentiation processes is not available yet.

Several studies on the effects of metal ions on cellular systems have been published in the past [20]. Concerning osseointegration metal ions were shown to play an important role in the processes of angiogenesis, osteogenesis, and mineralization of bone tissue. Above all, Cu(II)- and Co(II)-ions stimulate the secretion of certain growth factors, e.g., vascular endothelial growth factor (VEGF) via hypoxia inducible factor (HIF)-1, subsequently stimulating proliferation of endothelial cells and, thus, the formation of blood vessels [21]. The cations Zn(II) and Sr(II) instead stimulate osteoblast proliferation while inhibiting osteoclast activity [22,23]. These ions thus have a stimulating or anabolic effect on bone homeostasis. The cations Ca(II) and Mg(II) stimulate osteoblast proliferation. The latter also stimulates cell adhesion via binding of integrins to extracellular matrix proteins [24].

In this study, we investigated the effect of Fe-MEPE modified surfaces on the proliferation and differentiation of MG63 cells and BMSCs. As in our previous publication, the metallo-supramolecular coordination polyelectrolyte (MEPE) based on the ditopic ligand 1,4-bis (2,2′:6′,2′-terpyridin-4′-yl) benzene and Fe(II)-ions was used [1]. As several methods can be employed in order to deposit the positively charged Fe-MEPE on surfaces, we used the layer-by-layer deposition method and dip-coating from solution [25,26,27,28,29,30]. Due to the dynamic nature of the interaction of terpyridines and metal ions, such as Fe(II), Ni(II), or Co(II), MEPEs may represent ideal metal ion release systems. The released ions may stimulate specific cellular responses in target cells, which adhere to the modified surface.

## 2. Materials and Methods

### 2.1. Surface Modification

#### 2.1.1. Layer by Layer

Layer-by-layer coating was carried out using the method described earlier [27]. Circular white borosilicate glass slides with a diameter of 18 mm were washed with ethanol and dried with compressed air before. The polyelectrolytes were dissolved in aqueous 0.1 M sodium acetate solution to improve the layer thickness by weakening the intra-molecular electrical repulsion of the polymer chains [31]. First, a layer of polyethyleneimine (PEI, Fluka) was deposited on the glass slide using a coating solution with a concentration of 10^−2^ M. For the formation of the second layer, a 1 × 10^−3^ M poly-(styrene sulfonate) (PSS) solution was used. The final MEPE-layer was adsorbed using a 2.1 × 10^−3^ M Fe-MEPE solution. An incubation time of 4 minutes was chosen. Between the coating steps, the substrates were rinsed with ultrapure water. After the application of the last layer, the samples were dried with compressed air [27,29]. The resulting LbL sample consisted of a PEI, a PSS, and a final Fe-MEPE Layer.

#### 2.1.2. Dip Coating

Glass slides were washed with deionized water and finally with absolute ethanol. Fe-MEPE was dissolved in absolute ethanol with a concentration of 8.8 × 10^−3^ M [28]. Dip-coating was performed at ambient temperature by pulling the immersed glass slides at constant speed of 10 mm/min (Dip10) or 50 mm/min (Dip50) out of the coating solution. The slides were air-dried at ambient temperature.

### 2.2. Cell Culture and Biological Activity Testing

The MG63 (ATCC Number CRL-1427) and hMSCs (Donor 46: male, 73 years, Donor 54: female, 61 years, Donor 56: female, 75 years) cells were cultured in Dulbecco’s Modified Eagle’s Medium containing 10% FCS and 1% Penicilin/Streptomycin. For all experiments, 10,000 cells/cm^2^ were seeded onto the different substrates. Analysis of cell viability and -proliferation of the MG63 was performed 3 and 5 days after seeding. The hMSCs were analyzed after 2, 5, 7, and 9 days. To differentiate the two parameters, proliferation and metabolic activity, the cells were incubated with Dulbecco’s Modified Eagle’s Medium containing WST-1 (Roche). The metabolic activity was measured with an ELISA microplate reader (TECAN infinite M200) at 450 nm. After measurement, the cells were trypsinized and the cell number determined using a Neubauer counting chamber.

### 2.3. RNA Isolation and cDNA Synthesis

For RNA isolation, MG63 cells and hMSCs were trypsinized, subsequently washed with PBS, and centrifuged. RNA from the pelleted cells was isolated using the RNeasy Micro Kit (Qiagen, D-40724, Germany) according to the manufacturer’s instructions. RNA concentration and purity was determined by spectralphotometry at wavelengths of 260 nm and 280 nm, respectively. 500 ng of each RNA sample was reversely transcribed using the iScriptTM cDNA Syntheses Kit (BioRad, Hercules, CA, USA), according to the manufacturer´s recommendations.

### 2.4. Quantitative RT-PCR

Differentially expressed genes were quantified by qRT-PCR. The used primers are: hGAPDHsense, 5′-TGACGCTGGGGCTGGCATTG-3′ and hGAPDHantisense, 5′-GCTCTTGCTGGGGCTGGTGG-3′, ALPsense, 5′-CTTGACCTCCTCGGAAGACACTC-3′ and ALPantisense 5′-GCCTGGTAGTTGTTGTGAGCATAG-3′, Osteocalcinsense 5′-TTGGACACAAAGGCTGCAC-3′, and Osteocalcinantisense, 5′-CTCACACTCCTCGCCCTATT-3′.

PCR-reactions were carried out using a C1000TM Thermal Cycler including a CFX96 real-time monitoring system according to Belka et al. [1].

### 2.5. Colorimetric Determination of Iron(II)

The iron content of the cells was determined by a colorimetric assay based on ferrozine complexation. With this method it is possible to detect iron contents with a lower limit of 0.2 nmol. In brief, Fe(III)- and Fe(II)-ions have at first to be removed from proteins and the cellular network. For that the cells were washed thoroughly and lysed before the iron could be removed quantitatively from iron-loaded proteins like ferritin or heme-proteins. Subsequently, all iron species were reduced since ferrozine only forms complexes with iron (II). Cell lysis and complex formation by ferrozine is described in further studies [1].

## 3. Results

### 3.1. Preparation and Characterization of Modified Surfaces for Cell Growth

It is well understood that the physico-chemical properties of an interface greatly affect interactions with cells [32]. Therefore, meticulous attention is paid to the preparation and characterization of modified surfaces. The surface modification and characterization follows the experimental protocol established in a previous study [1]. Two methods of surface modifications are employed for the current study. First, the layer by layer deposition is used to deposit the metallo-polymer on the surface [27,29,33]. The final films are composed of a polyethyleneimine primer, poly-(styrene sulfonate) (PSS), and finally Fe-MEPE. Second, Fe-MEPE is directly applied from solution onto the substrate by dip-coating [28]. The final layer thickness amounts to 25 ± 1 nm (FeDIp10) and 43 ± 2 nm (FeDip50). The roughness of the modified surfaces is in the range of 3–7 nm [1]. The mean values of the contact angles are 69 ± 2° for the Dip10 and 67 ± 2° for the Dip50 substrates, indicating that both substrates are hydrophilic (data not shown) [34]. The contact angle of the LbL surface is 57 ± 2° [1].

### 3.2. Cell-Substrate Interactions

**Figure 1:**
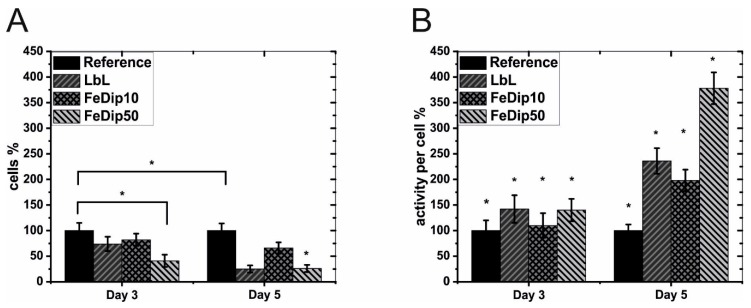
(**A**) Cell number and (**B**) metabolic activities of the used MG63 cells was determined at day 3 and 5 for the particular surface modifications. The cell number and the metabolic activity were set in relation to cells grown on tissue culture polystyrene (TCPS) substrates (reference). The experiments were performed at least two times in triplicate. * *p* < 0.5 (analyzed by one-way ANOVA with Tukey test).

First, we investigated the cell number and the metabolic activity of the cell line MG63 to assess the cytotoxicity of Fe-MEPE in relation to the TCPs-reference (tissue culture polystyrene) surface. In Figure 1, cell number (A) and activities (B) of MG63 cells grown on the functionalized surface are shown for day 3 and 5. 

As can be seen, application of Fe-MEPE modified surfaces had a significant influence on both, cell number and cell activity of the used osteosarcoma cells. At both days, the evaluated cell number for the functionalized substrates was below those of the reference substrate. The activity of the FeDip10 modified surface was 82 ± 12%, which is within the non-cytotoxicity limit defined for the range of 81–100%. In the case of the FeDip50 layer, a significantly reduced proliferation at day 3 can be observed, thus classifying this surface as cytotoxic with a degree of 3 [35]. Relative to the reference substrate, the cell number for the FeDip50 modified surface dropped from 41 ± 12% at day 3 to 26 ± 7% at day 5. The relative cell number on the LbL-modified surface decreased similarly from day 3 to day 5. Here, the values decreased from originally 74 ± 14% to 25 ± 7%. The FeDip10 modified surfaces showed moderately reduced cell proliferation from day 3 to 5, with values ranging from 82 ± 12% to 66 ± 11% compared to the reference substrate.

**Figure 2:**
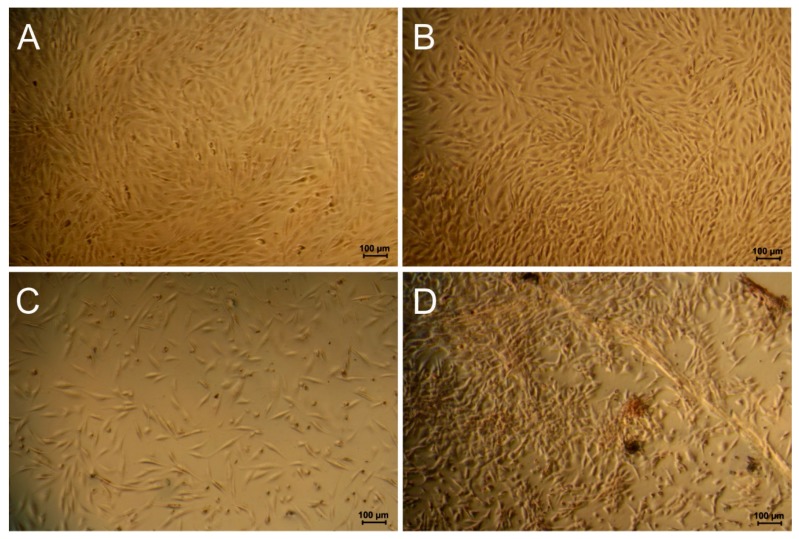
Light microscopy images of Alizarin red stained MG63 cells. (**A**) Cells grown on Reference substrate, (**B**) LbL, (**C**) FeDip10, and (**D**) FeDip50 at day 3.

Comparing the cell activity on the basis of the mitochondrial activity per cell Figure 1B shows that the cells stopped proliferation in favor of metabolic activity, reaching 378 ± 31% at day 5 for FeDip50 modified surfaces compared to cells grown on the TCP reference surface. Cells grown on LbL modified surfaces showed an increase in metabolic activity of 139 ± 2 5% and FeDip10 modified surfaces of 98 ± 21%, respectively. These high metabolic cell activities suggest that cells grown on Fe-MEPE modified surfaces might have been stimulated to differentiate most likely to the osteogenic lineage. To confirm this hypothesis, alizarin red staining was carried out in order to detect a potentially increased Ca(II)-ion storage in the cells. Figure 2 shows microscopy images of the alizarin red stained cells at day 3. 

The overall morphology and the cell number of cell grown on the reference substrates differed greatly as seen in the images taken from the individual surfaces. On the FeDip10 modified surfaces cells did not reach confluency, while cells grown on the reference substrate appeared confluent.

**Figure 3:**
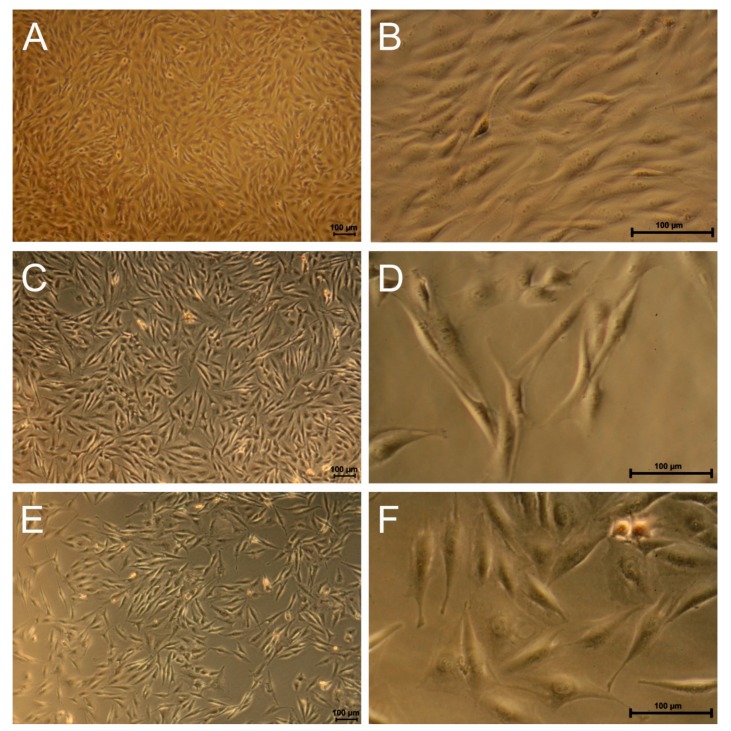
Light microscopy image**s** at different magnification of Alizarin red stained MG63 cells grown on (**A**,**B**) reference substrates, (**C**,**D**) FeDip10, and (**E**,**F**) on FeDip50 substrates at day 5.

The low cell numbers are better visualized in Figure 3, showing Alizarin red stained cells at two different magnifications at day 5.

Based on cell morphology, cluster formation correlating with Alizarin red staining indicates osteogenic differentiation on the FeDip50 modified surfaces already on day 3, which represents the second differentiation stage according to Collin et al. [12].

By detachment and reseeding after growth for three days on Fe-MEPE modified surfaces, the already-formed clusters were disrupted, and thus, the cells had to re-initiate themselves. Due to the very low relative proliferation rates of the MG63 cells on Fe-MEPE modified substrates (see Figure 1) only small cell deposits on dip-coated Fe-MEPE modified surfaces can be seen at day 5. However, cells significantly differing in the overall morphology can be observed (Figure 3F), which were also characterized by increased Ca(II)-ion deposition (Figure 2D). On all Fe-MEPE modified surfaces, the cells adhere strongly with extended cell protrusions. As expected and in agreement with previous experiments, MG63 cells differentiate according to their initial origin (osteogenic) on Fe-MEPE modified surfaces [1]. In order to determine whether the used Fe-MEPE modified surfaces support in general differentiation process of already committed cells, we expanded our experiments by including human primary cells, so-called bone marrow derived mesenchymal stem cells (BMSCs) obtained from the spongiosa of femur bones.

**Figure 4:**
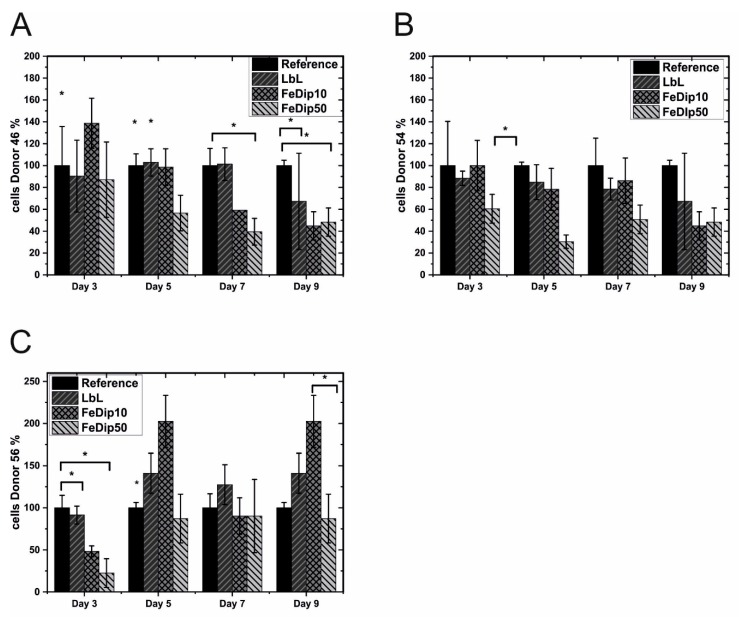
Cell growth of the used bone derived mesenchymal stem cells (BMSCs) from (**A**) Donor 46, (**B**) Donor 54, and (**C**) Donor 56 relative to that on the reference surface for the particular surface modifications at four different time points. All experiments were performed in triplicate. * *p* < 0.5 (analyzed by one-way ANOVA with Tukey test).

**Figure 5:**
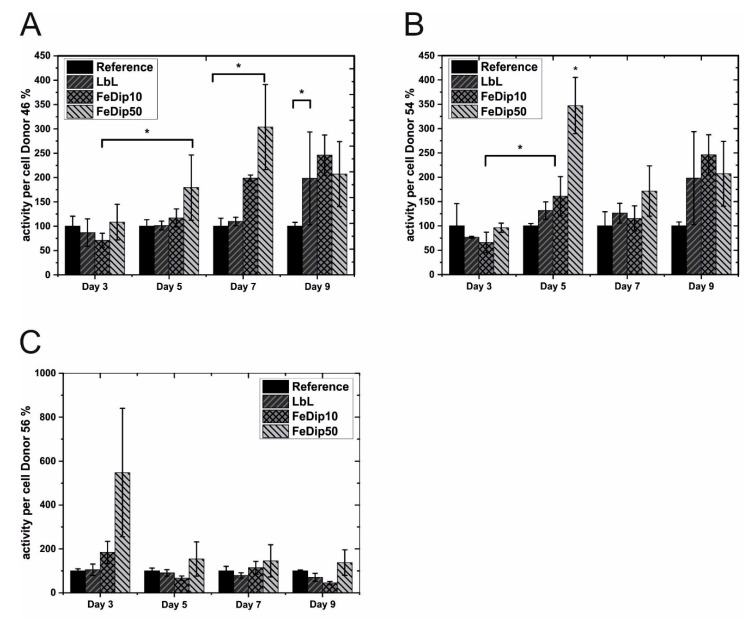
Relative Metabolic activities of the used hMSC cells from (**A**) Donor 46, (**B**) Donor 54, and (**C**) Donor 56 was determined at four different time points for the particular surface modifications in relation to cells grown on reference substrates. All experiments were performed in triplicate. * *p* < 0.5 (analyzed by one-way ANOVA with Tukey test).

As in the previously reported experiments, BMSCs are seeded and grown for up to 9 days on LbL, FeDip10 and FeDip50 modified surfaces. As in Figure 1 and Figure 2, cell count and cell activity of BMSCs derived from three donors which are grown on LbL, FeDip10 and FeDip50 modified surfaces as shown in Figure 4 and Figure 5. 

In general, similar results were obtained regarding proliferation of cells obtained from donor 46 and 54 on the differently modified surfaces compared to the reference surface. As shown for MG63 cells above, we note here the most prominent reduction in cell growth of 56 ± 16% for cells (donor 46) grown on FeDip50 modified substrates compared to the reference substrate. For donor 54, a reduction to 60 ± 13% compared to the reference substrate is observed. A reduction of proliferation for cells grown on LBL-modified surfaces as shown for MG63 cells could not be observed. In contrast, cells of donor 56 appear to proliferate much more on LBL- and Dip10-modified surfaces, whereas cell growth on FeDip50-modified surfaces is comparable to that of the reference substrate. Cells of this donor thus seem to differentiate less than cells of donor 46 and 54.

In order to proof the above mentioned hypothesis, the cell activity is tested by WST-1 assays. On the one hand, the observed low cell numbers might be caused by cell death or, on the other hand, are the result of a proliferative stop, which typically accompanies differentiation processes. If the cells are simply not viable due to a lack of cell adhesion or to a release of cytotoxic substances from the particular MEPE-modified surfaces, no increase in metabolic activity should be observed in these assays. As shown in Figure 5, the determined metabolic activity depicted as mitochondrial activity per cell, argue against cell death since values obtained from cells grown on the MEPE modified surfaces are throughout the duration of the experiment at least equal to those determined for cells grown on the reference substrates. For samples employing cells from donor 46 and 54 a clear trend towards an increased cell activity is apparent from day 5. At day 5, the relative activity of cells from donor 46, which are grown on FeDip50 modified substrates, are found to be 180 ± 67% compared to the reference surfaces. For cells derived from donor 54 this value rises to over 300% of that of the reference. In contrast, for cells grown on LbL-modified substrates the metabolic activities are not increased compared to the reference.

Thus, based on these observations, we assume that cells, which are grown on the dip-coated surfaces, tend to differentiate. Due to the osteogeneous origin of the used BMSCs, a differentiation towards osteogenic lineages is expected, which can be assessed by specific assays such as Alizarin red staining (as shown for MG63 cells, see Figure 3) or by determination of alkaline phosphatase (ALP) activity.

**Figure 6:**
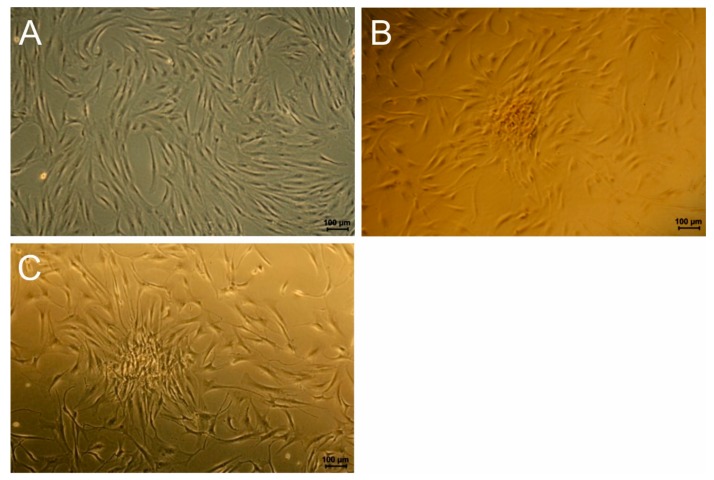
Examples of light microscopy images of Alizarin red stained BMSCs grown on (**A**) reference (Donor 409 46), (**B**) FeDip50 (Donor 46), and (**C**) FeDip50 (Donor 54) at day 5.

Figure 6 shows microscopy images of Alizarin red stained cells of donor 46 and 54 on the reference and FeDip50 surface at day 5. 

It becomes obvious that the cell morphology on the reference has not changed. Importantly, no staining by Alizarin red is detectable indicating that these cells reside in an undifferentiated state, which supports the data obtained for the cell activity (see Figure 5). In clear contrast, cells grown FeDip50 modified surfaces exhibit a different spindle-like phenotype. The cells are assembled in form of clusters, which are positively stained by Alizarin red. The cells on the reference surface appear denser than those grown on dip coated surface. This observation is in agreement with the determined cell numbers shown in Figure 4. Based on these stainings we conclude that the cells grown on FeDip50 modified surfaces have reached after stage 2 5 days differentiation in accordance with Collin et al. [12]. Thus, altered cellular activity and cell morphology indicate differentiation of the used cells towards the osteogenic lineage as supported by the positive alizarin red staining.

### 3.3. Iron Content

**Figure 7:**
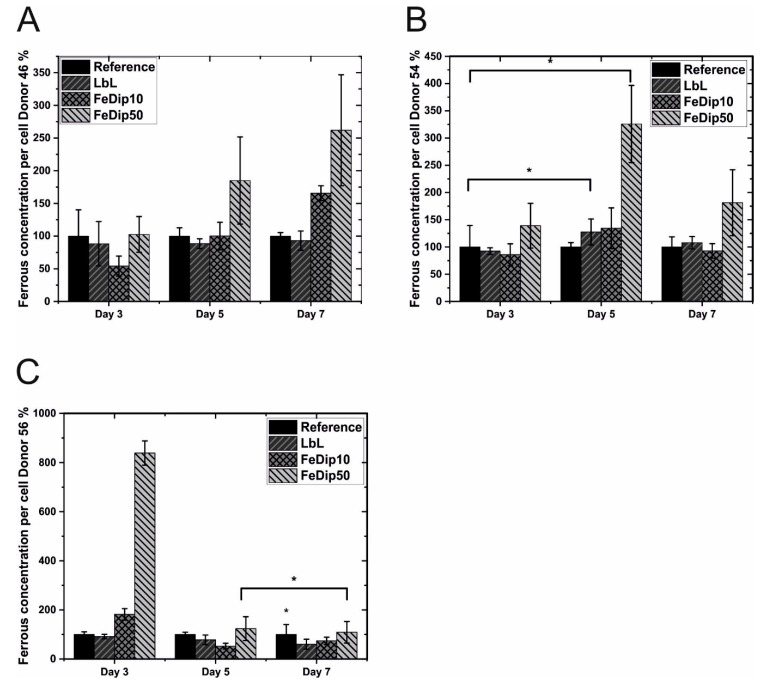
The bar diagrams represent the relative ferrous concentrations per cell for (**A**) donor 46, (**B**) donor 54, and (**C**) donor 56 grown on the indicated substrates. The values obtained for cells grown on TCPS substrates are set to 100% (the values for the references substrates also vary donor dependently. The absolute values for the reference substrates at day 3 are: donor 46 = 68.2 fMol/cell; donor 54 = 31 fMol/cell; donor 56 = 21.6 fMol/cell). All experiments were performed in triplicate. * *p* < 0.5 (analyzed by one-way ANOVA with Tukey test).

In order to show whether potential differences in the intracellular iron content may depend on the particular surfaces, the cells are removed from the substrates, washed, lysed, and the iron ions released from the carriers such as ferritin and hem-proteins are detected by photometric analysis. The relative content of iron ions of cells grown on the particular Fe-MEPE surfaces compared to the reference surface are shown in Figure 7.

In agreement to the data concerning cell number and activity, cells from donor 46 and 54 grown on Fe-MEPE modified surfaces show, as expected, an increased intracellular iron ion content over a prolonged time of 9 days compared to the reference. On the other hand, cells from donor 56 show an initial increase in iron ion content on day 3; however, starting from day 5, no significant differences in iron ion content compared to the control could be detected.

The influence of certain metal ions on BMSCs has already been shown. For instance, Yoshizawa et al. detected a rapid proliferation of BMSCs and an increase in extracellular matrix (ECM) mineralization in the presence of Mg(II)-ions in vitro [36]. On the other side, transition metal ions such as Mn(II), Fe(III), Co(II), Ni(II), and Cu(II), showed cytotoxic effects on osteoblastic cell lines such as MG63 cells already at a concentration of 0.1 mM [37]. Typically, osteogenic differentiation of BMSCs is initiated by addition of various agents such as dexamethasone, L-ascorbic acid-2-phosphates or ascorbic acid and β-glycerophosphates. As the aforementioned agents are absent in the experiments shown here, we assume that solely the Fe-MEPE modified surfaces trigger differentiation of the used BMSCs. However, the determination of the concentration of the active species acting on the cell in the interfacial region remains to be elucidated. As positive staining by Alizarin red already indicates differentiation of the investigated BMSCs towards osteogenic lineages this process is analyzed in more detail by investigating the expression profiles of characteristic marker genes such as ALP and Osteocalcin (OC) by qRT-PCR.

### 3.4. Quantification of ALP and OC Gene Expression

BMSCs, when grown for 16 days in the aforementioned differentiation media, form a coherent network of ALP-positive cells, but when Co(II)-ions at a concentration of 40 μM to 100 μM are added, a decrease in the overall ALP activity is observed [38,39].

**Figure 8:**
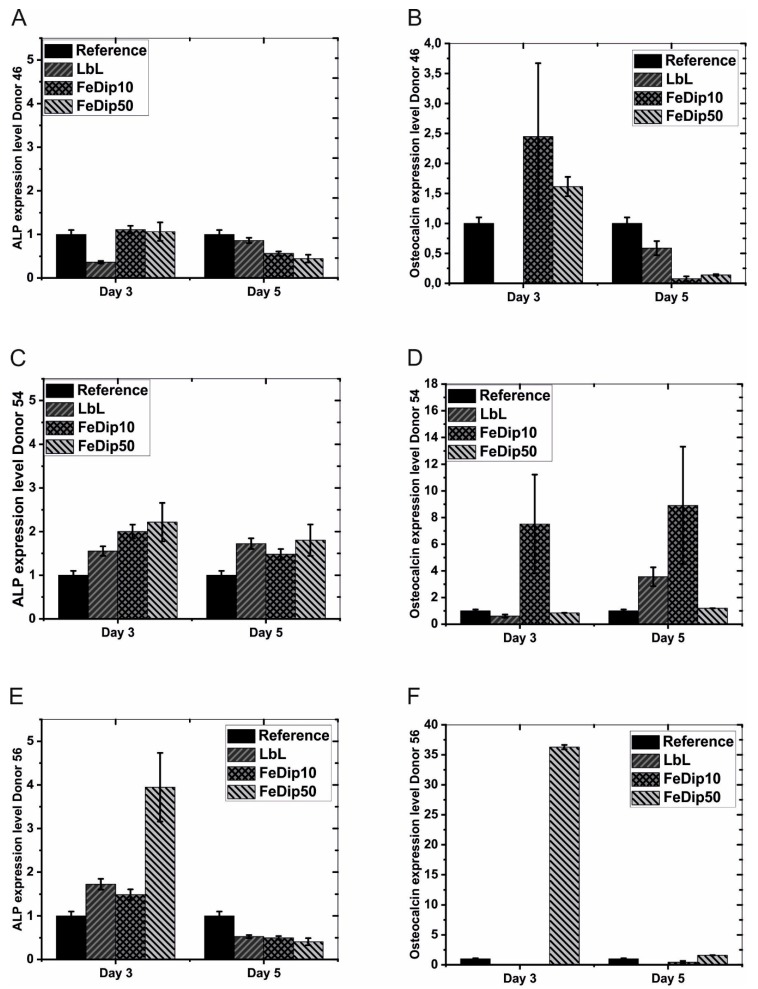
The bar diagrams represent values for relative ALP (**A**,**C**,**E**) and OC (**B**,**D**,**F**) expression levels of cells grown on the indicated substrates of Donor 46 (**A**,**B**), Donor 54 (**C**,**D**), and Donor 56 (**E**,**F**). All values are normalized to GAPDH expression levels. Subsequently the levels of ALP and OC expression determined for cells grown on reference substrates are set to 1. All experiments were performed in triplicate.

Figure 8 shows ALP and OC gene expression at day 3 and day 5 of the three donors.

In contrast to the clear results obtained from experiments regarding cell number and cell activity those addressing ALP and OC expression need to be described and discussed in more detail. Unexpectedly, ALP expression in cells of donor 46 grown on FeDip modified surfaces is in general comparable to that of cells grown on the reference substrates. At day 5, the values for ALP activity decrease by half relative to the reference. For the LbL modified surface lower ALP expression is observed at day 3, but it rises almost to the reference value by day 5.

Since these findings are not in agreement with our data regarding cell number and activity, we also analyzed the expression of OC. Here, a significant increase in OC expression is observed for cells of donor 46 grown on FeDip10 and FeDip50 modified surfaces at day 3 if compared to the reference substrate. However, by day 5 OC expression in these cells declines strongly to values of approximately 10% of those of the reference. Instead, cells of donor 54 grown on the Fe-MEPE modified surfaces in general show higher ALP expression levels if compared to the controls. Astonishingly, cells of donor 56 show high expression of either ALP and OC but only if grown on FeDip50 modified surfaces at day 3, which is unexpected since data concerning cell number and activity identify cells of this donor to be the least differentiating ones.

Taken together, the results of these experiments suggest that Fe-MEPE-triggered differentiation strongly depends on the individual surface modification and, as expected, also on the individual donor thus preventing an exact determination of particular differentiation stages. Alkaline phosphatase, as already mentioned in the introduction, is primarily expressed upon osteogenic differentiation in two separated time frames. A first peak is observed at the beginning of osteogenic differentiation and a second at the beginning of mineralization. As shown by individual microscopy images, only sub-populations of cells can be stained by alizarin red implicating that cell differentiation, even in one specified sample, is non-homogenous. Thus, the data obtained from qRT-PCR experiments, representing the average ALP or OC expression levels of these non-homogenously differentiated cells, may not indicate a general differentiation stage.

However, it can be assumed that differentiation of at least some of the cells of donor 46 reached the second differentiation step already at day 3, which is confirmed by alizarin red staining (see Figure 8) but a comprehensive progress in differentiation till day 5 cannot be detected

Collin et al. observed an increase in osteocalcin expression by 30-fold during mineralization [12]. This considerable increase in osteocalcin gene expression might be achieved for BMSCs of donor 56 simply grown for 3 days on FeDip50-modified surfaces. Birgani et al. reported no increased ALP or osteocalcin gene expression until day 14 after metal ion addition. Moreover, Birgani et al. suggests that the ALP activities of the different donors can vary [40]. These different activities as a function of the BMSC donor have already been first described by Barbara et al already in 2004 but meanwhile also by many others [41,42,43,44,45]. The variety of gene expression between the different donors has also been observed in our study. But the duration of ALP and OC gene expression was reduced from 14 to only 3 days.

However, the results presented here indicate that osteogenic differentiation is initiated by Fe-MEPE modified surfaces and can be detected at day 3. In this study we detect ALP expressing cells already at day 5. Thus, the use of Fe-MEPE modified surfaces seems to trigger the osteogenic differentiation processes more rapidly.

## 4. Conclusions

The current investigation employing MG63 cells grown on Fe-MEPE modified substrates suggest initiation of osteogenic differentiation by both, high cell activity and altered morphology of the cells and/or cluster formation. Remarkably, LbL and FeDip50 modified surfaces show strongest effects on cell count and cell activity, which becomes visible already at day 3. Considering morphology, cells grown on LbL modified surfaces appear morphological similar and do not form cell clusters like those grown on the reference substrate. Based on these observations, MG63 cells seem to best differentiate to osteoplastic lineages if grown on these substrates, which is further supported by the occurrence of Alizarin red stained clusters (Figure 2).

Concerning BMSCs, our findings suggest that Fe-MEPE modified surfaces also stimulate osteogenic differentiation in these cells. Similar to MG63 cells, the Fe-MEPE modified surfaces suppress proliferation and promote differentiation of the used BMSCs to a variable extent which is dependent on the individual donor. Cells derived from two donors (46/54) differentiate better than cells of the third donor (56), which is supported by staining with Alizarin red. Clearly, BMSCs are stimulated for osteogenic differentiation, which appears donor and substrate specific. However, cell differentiation occurs non-coherently thus reflecting various differentiation stages already in one particular sample.

The effect of the modified surfaces on osteogenic differentiation can also be detected on mRNA level addressing well-known osteogenic marker genes, such as ALP and OC, although the data do not show the expected coherency. Nevertheless, all Fe-MEPE modified surfaces, which are investigated here, influence the osteogenic differentiation capacity of BMSCs without addition of agents inducing osteogenic differentiation. This remarkable result leads to the question of the underlying mechanism. It is well established, that metal ions affect cell differentiation and, therefore, most likely also a release of iron ions into the interfacial contact region between substrate and cells initiate cellular processes towards differentiation. The osteogenic properties of iron ions in context of BMSC differentiation has also been reported for iron oxide nanoparticles by Wang et al. [46,47]. Thus, our results indicate that Fe-MEPE functionalized surfaces may serve as innovative scaffolds for the treatment of bone defects.
